# First functional evidence that a rare germline *TP53*β variant drives senescence-associated immune suppression and impairs apoptosis and cell migration in breast cancer patients

**DOI:** 10.1016/j.tranon.2025.102616

**Published:** 2026-01-06

**Authors:** Claudia Christowitz, Daniel W Olivier, Nicole van der Merwe, Maritha J Kotze, Anna-Mart Engelbrecht

**Affiliations:** a Department of Physiological Sciences, Faculty of Science, Stellenbosch University, Stellenbosch, South Africa; b Division of Chemical Pathology, Department of Pathology, Faculty of Medicine and Health Sciences, Stellenbosch University, Cape Town, South Africa; c Division of Chemical Pathology, Department of Pathology, National Health Laboratory Service, Tygerberg Hospital, Cape Town, South Africa; d African Cancer Institute, Department of Global Health, Faculty of Medicine and Health Sciences, Stellenbosch University, Cape Town, South Africa

**Keywords:** *TP53*, Cellular senescence, Functional genomics, Personalized medicine, Breast cancer

## Abstract

**Introduction:**

Pathology-supported genetic testing (PSGT), a personalized medicine framework established in South Africa, led to the identification of a rare germline *tumour suppressor protein 53* (*TP53*) beta-isoform (β) variant (NM_001126114.3, c.1018A>*G*, p.N340D) in a family with the Li-Fraumeni-like syndrome. While protein modeling predicted structural alterations consistent with impaired function, its pathogenicity remained unclear.

**Aim:**

To determine the functional impact of the *TP53*β N340D variant on cell proliferation, cell death, senescence, migration, and anti-tumour activity using a translational *ex vivo* model.

**Methods:**

Peripheral blood mononuclear cells (PBMCs) were isolated from female controls and breast cancer patients, with or without the *TP53*β N340D variant. Lipopolysaccharide (LPS)- and phytohemagglutinin-L (PHA-L)-stimulated proliferation was evaluated by a water-soluble tetrazolium 1 (WST-1) assay. Doxorubicin (DXR)-induced cell death was assessed using a WST-1 assay, flow cytometry, and western blotting. A senescence-associated beta-galactosidase assay and western blotting determined senescence. Migration and anti-tumour activity were assessed using a Transwell assay and co-culturing PBMCs with BT-549 spheroids.

**Results:**

The *TP53*β N340D variant impaired DXR-induced cell death (*p* < 0.001), supported by reduced late apoptosis and decreased CASP3 and PARP activation. *TP53*β N340D PBMCs exhibited increased senescence (*p* < 0.01), potentially contributing to chemoresistance. Reduced LPS- and PHA-L-stimulated proliferation was dependent on cancer status. The variant reduced PBMC migration (*p* < 0.01), suggesting altered immune recruitment. Although anti-tumour activity appeared reduced in *TP53*β N340D PBMCs, spheroid size remained unchanged.

**Conclusions:**

This study provides supporting evidence for the pathogenicity of the *TP53*β N340D variant and highlights the importance of integrating functional genomics into PSGT to enhance medical decision-making.

## Highlights

• Germline *TP53*β N340D variant disrupts peripheral blood mononuclear cell function.
• *TP53*β N340D variant increases cellular senescence and impairs apoptosis and migration.
• Functional genomics provides evidence for rare germline variant classification.
• Pathology-supported genetic testing utilizes functional genomics for interpretation.
• First *ex vivo* evidence supporting the pathogenicity of the *TP53*β N340D variant.

## Introduction

Breast cancer was the second most common cancer and the fourth leading cause of cancer death in 2022, with 2.3 million new cases and 666,000 deaths globally [1]. Despite decades of research, cancer remains complex and not fully understood. Key hallmarks of cancer include sustained proliferative signalling, evasion of growth suppressors, resistance to cell death, replicative immortality, invasion and metastasis, angiogenesis, immune evasion, and altered cellular energetics, enabled by tumour-promoting inflammation and genome instability [2,3].

Given the heterogeneity of breast cancer, traditional one-size-fits-all treatment approaches are often inadequate. This necessitates the development of innovative strategies such as pathology-supported genetic testing (PSGT), which provides a personalized medicine framework for tailoring treatment to the unique characteristics of each tumour and individual [4]. The PSGT approach facilitates differentiation between inherited, lifestyle-triggered, and therapy-induced pathologies [5,6]. Christowitz et al. (2024) recently highlighted the need for the incorporation of functional studies into this framework to help assess the pathogenicity of variants of uncertain significance (VUS) detected by next-generation sequencing (NGS) [7]. Given the clinical interpretation challenges imposed by the detection of rare novel variants, these authors identified *ex vivo* functional genomics studies as a feasible experimental approach to increase access to personalized medicine in Africa, supported by the Open Genome Project, an integrated service and research program [6,7]. This initiative enabled the current study which focused on the clinical significance of the *tumour suppressor protein (TP53)* beta-isoform (β) variant, NM_001126114.3, c.1018A>*G*, p.N340D, described by Leroy et al. (2017) in tumour-extracted DNA [8]. This variant has been reported twice as a somatic variant in the *TP53* online database of >80 000 tumours.

Kotze et al. (2019) detected the *TP53* N340D variant in the germline DNA of a South African patient diagnosed with three primary invasive breast carcinomas between the ages of 30 and 60 years [9]. Prior to genetic testing using whole exome sequencing (WES), the patient received radiotherapy which is a contraindication for germline pathogenic *TP53* variants. The oncologist who referred the patient for PSGT was concerned that the treatment regimen contributed to the development of subsequent cancers. Further family screening using Sanger Sequencing confirmed co-segregation of the *TP53* N340D variant with cancer on the maternal side of the family. As deleterious *TP53* variants may exhibit variable penetrance within families, radiation exposure implied potential additive gene-environment risk. Future clinical decision-making in this family should therefore consider the risks of normal tissue toxicity and tumour response to radiotherapy.

This study determined the *ex vivo* functional impact of the *TP53*β N340D variant initially predicted as deleterious by *in silico* tools, as supported by protein modelling displaying altered structural changes suggesting impaired protein function [10]. The variant is absent in population databases such as gnomAD, and no other potential pathogenic variants were detected in moderate-to-high-risk cancer susceptibility genes using multiplex ligation-dependent probe amplification and targeted NGS [11], as well as whole genome sequencing [12]. Genetic counselling offered to the extended family with multiple cancers furthermore uncovered the Li-Fraumeni-like phenotype. These findings substantiated a consensus decision by the Scientific and Technical Advisory Panel of the Open Genome Project to classify the *TP53*β N340D variant as likely pathogenic. In alignment with the initiative’s commitment to returning clinically meaningful research results to participants, this study aimed to investigate the functional impact of the *TP53*β N340D variant on cell proliferation, cell death, senescence, migration, and anti-tumour activity using a translational *ex vivo* model.

## Methods

### Study participants and sample collection

Female participants were enrolled in the study following genetic counselling conducted as part of the informed consent process. Two *TP53*β N340D variant-positive controls and two breast cancer patients carrying the *TP53*β variant were included, in addition to 11 controls and 11 breast cancer patients without the variant [*TP53*β wild type (WT)]. All breast cancer patients were in remission and not undergoing active anti-cancer treatment. Blood samples were collected in EDTA tubes (BD Biosciences, US) for DNA extraction and citrate tubes (BD Biosciences, US) for peripheral blood mononuclear cell (PBMC) isolation.

### Genotyping

Genomic DNA was extracted from whole blood using the Chemagic™ DNA Blood 400 Kit H96 (Revvity, US) on the Chemagic™ 360 automated platform (Revvity, US), and stored at −20 °C. Polymerase chain reaction (PCR) was performed using Phusion™ Plus PCR Master Mix (Thermo Fisher Scientific, US) and products were stored at −80 °C. Post-PCR clean-up was performed with a NucleoFast 96-well plate (Macherey-Nagel, DE) on a Tecan EVO 150 robotic workstation (Tecan Life Sciences, CH). Sanger sequencing was conducted using the BigDye Terminator v3.1 Cycle Sequencing Kit (Thermo Fisher Scientific, US) and run on an ABI 3730xl DNA Analyzer (Thermo Fisher Scientific, US), according to the manufacturer’s protocol. Alignment was performed with Clustal W.

### PBMC isolation and culturing

PBMCs were isolated by density centrifugation using Histopaque®−1077 (Sigma-Aldrich, US). Following centrifugation and washing, cells were counted and resuspended in heat-inactivated FBS (HI-FBS) with 10 % DMSO for cryopreservation at −80 °C for 24 h and stored in liquid nitrogen. PBMCs were cultured in RPMI 1640 GlutaMAX™ medium with 10 % HI-FBS and 1 % penicillin-streptomycin (Thermo Fisher Scientific, US), seeded in non-treated flasks, and incubated at 37 °C with 5 % CO₂. After 24 h, cells were harvested with StemPro™ Accutase™ (Thermo Fisher Scientific, US), seeded, and incubated for 4 h before treatment.

### WST-1 assay

PBMCs (∼300,000 cells/cm²) were seeded in 96-well plates and treated for 72 h with 10 µg/mL lipopolysaccharide (LPS), 1 µg/mL phytohemagglutinin-L (PHA-L), 1 µM doxorubicin (DXR), 185 µM 5-fluorouracil (5-FU), 65 µM etoposide, or 15 µM gemcitabine (GEM) (Sigma-Aldrich, US). WST-1 reagent (Merck, DE) was added at 70 h and incubated for 2 h. Absorbance at 450 nm was measured using a Synergy™ HTX Multi-Mode Microplate Reader (Agilent, US). Data were analysed with Kruskal-Wallis and Dunn’s post-test (*TP53*β WT, *N* = 11; *TP53*β N340D, *N* = 2).

### Flow cytometry

PBMCs (∼250,000 cells/cm²) were treated with 1 µM DXR for 72 h in T25 flasks, harvested, stained with Annexin V Alexa Fluor® 488 and propidium iodide (PI) in Annexin V-binding buffer (Thermo Fisher Scientific, US), and analysed on a FACS Melody™ Cell Sorter (BD Biosciences, US). Sphero™ Rainbow Calibration Particles (BD Biosciences, US) were used for standardization, and compensation was performed using 10 % DMSO-treated PBMCs. Data were analysed in FlowJo v10.8.1 and two-way ANOVA with Tukey’s post-test was performed (*TP53*β WT, *N* = 8; *TP53*β N340D, *N* = 2).

### Western blot analysis

PBMCs (∼160,000 cells/cm²) were treated with 1 µM DXR for 72 h in T75 flasks. After harvesting, lysates were sonicated in RIPA buffer, quantified using Bradford assay, and denatured with Laemmli buffer. Proteins (20 µg) were resolved on 10 % Stain-Free™ gels (BioRad, US) and transferred to PVDF membranes on the Trans-Blot® Turbo™ Transfer System (BioRad, US). Membranes were blocked, probed with primary and secondary antibodies (**Table A.1**), and detected with Clarity™ ECL substrate on the ChemiDoc™ MP System (BioRad, US). Total protein was used for normalization using Image Lab™ software (**Figure A.1-A.10**). Three-way ANOVA with Sidak’s test allowed consolidation of disease state, enabling two-way ANOVA with Tukey’s post-test (*TP53*β WT, *N* = 16; *TP53*β N340D, *N* = 4).

### Senescence-associated β-galactosidase assay

PBMCs (∼250,000 cells/cm²) were treated with 1 µM DXR for 72 h in 24-well plates and stained with the SA-β-gal kit (Cell Signaling Technology, US). Fixed cells were incubated overnight in non-CO_2_ with staining solution, overlaid with 70 % glycerol, imaged, and quantified using ImageJ. Data were analysed using three-way ANOVA with Sidak’s test (*TP53*β WT, *N* = 2; *TP53*β N340D, *N* = 2).

### Transwell migration assay

PBMCs (∼300,000 cells/cm²) were seeded in the upper chamber of 8 µm-pore Corning® HTS Transwell® plates (Merck, DE) in serum-free RPMI. Lower chambers contained RPMI with or without 10 % HI-FBS. After 24 h, adherent migrated cells on the underside of the inserts were fixed and stained with 0.2 % crystal violet (Sigma-Aldrich, US). Adherent and non-adherent cells were imaged and quantified using ImageJ. Data were analysed with three-way ANOVA with Sidak’s test (*TP53*β WT, *N* = 2; *TP53*β N340D, *N* = 2).

### Spheroid co-culture assay

*Co-culturing:* BT-549 cells (5000 cells/well) were seeded in Nunclon™ Sphera™ 96-well U-bottom plates (Thermo Fisher Scientific, US), centrifuged, and incubated in RPMI 1640 medium (Thermo Fisher Scientific, US) with 10 % FBS and 10 μg/mL insulin. On day 5, BT-549 spheroids were co-cultured with PBMCs (25 000 cells/well) for four days. Images were acquired daily, and spheroid size was quantified using ImageJ. Data were analysed using mixed-effects model with Tukey’s test (*TP53*β WT, *N* = 3; *TP53*β N340D, *N* = 2).

*Immunocytochemistry:* Co-cultured spheroids were fixed, permeabilized, blocked, and probed with a CD45 antibody and Alexa Fluor® 488 (**Table A.1**), and Hoechst counterstaining (1:200, Thermo Fisher Scientific, US). Fluorescence imaging was conducted using a Zeiss LSM 780 Elyra AxioObserver Confocal Microscope (Zeiss, DE).

### Statistical analyses

Statistical analyses were performed using GraphPad Prism® v9.3.1. Outliers were identified, followed by normality and lognormality tests. Appropriate parametric (mean ± SEM) or nonparametric (median with IQR) tests, along with recommended multiple comparisons tests, were conducted and described in each section. A p-value <0.05 was considered statistically significant.

## Results

### Genotyping

**Fig. 1:**
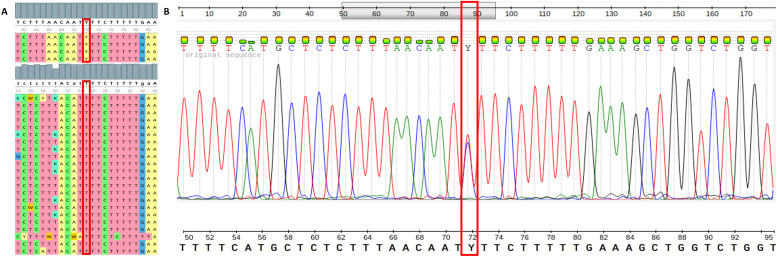
**(A)** Sanger sequence results illustrate the presence (*Y* = *C*/T) or absence (T/T) of the *TP53*β N340D variant (c.1018A>*G*, p.N340D) in all study participants. **(B)** Representative electropherogram of Sanger sequencing data indicating two distinct signals at Y (C/T) at position 72 of the amplicon in a heterozygous carrier of the *TP53*β N340D variant. *TP53*: tumour suppressor protein 53.

Genotype analysis revealed four heterozygous *TP53*β N340D carriers (*Y* = *C*/T, amplicon position 72) and 22 *TP53*β WT individuals (T/T, amplicon position 25) (Fig. 1A). Heterozygosity was confirmed in electropherograms with peaks showing two distinct signals (Fig. 1B). In contrast, homozygous peaks were identified as sharp, well-defined single peaks of consistent height.

### Cell proliferation and death

#### WST-1 assay

**Fig. 2:**
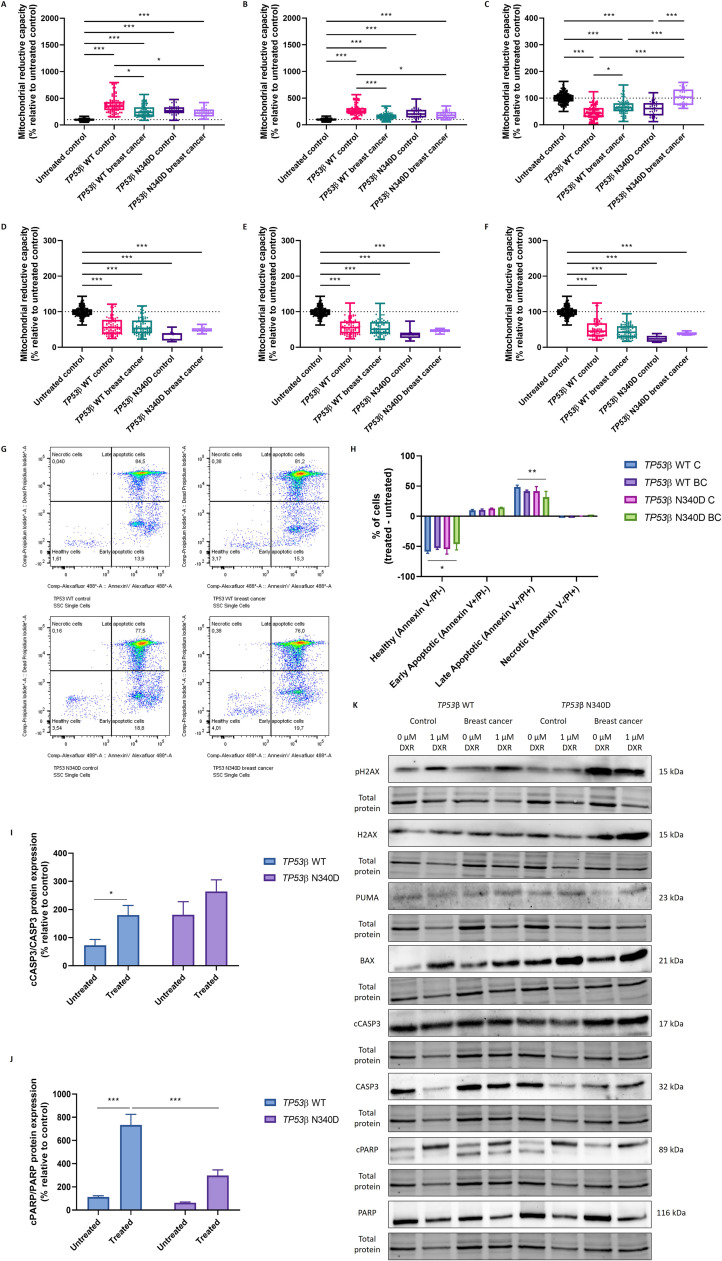
Mitochondrial reductive capacity, as a measure of cell proliferation/death, of PBMCs, isolated from *TP53*β WT and *TP53*β N340D control individuals and breast cancer patients, treated with 10 µg/ml lipopolysaccharide **(A)**, 1 µg/ml phytohemagglutinin-L **(B)**, 1 µM DXR **(C)**, 185 µM 5-fluorouracil **(D)**, 65 µM etoposide **(E)**, or 15 µM gemcitabine **(F)** for 72 h. Data are presented as median with IQR (*N* = 2–11). * *p* < 0.05; *** *p* < 0.001. Flow cytometric analysis of PBMCs, isolated from *TP53*β WT and *TP53*β N340D control individuals and breast cancer patients, following 1 µM DXR treatment for 72 h. Cells were stained with Annexin V-Alexa Fluor® 488 and PI and subsequently analysed on a BD FACS Melody™ Cell Sorter. **(G)** Representative flow cytometry density plots indicating the percentage of cells in each quadrant, namely Q1 (necrotic cells), Q2 (late apoptotic cells), Q3 (healthy cells), and Q4 (early apoptotic cells). **(H)** Percentage of healthy (Annexin V-/PI-), early apoptotic (Annexin *V*+/PI-), late apoptotic (Annexin *V*+/PI+) and necrotic (Annexin V-/PI+) cells in each group. Data are presented as mean ± SEM (*N* = 2–8). * *p* < 0.05; ** *p* < 0.01. Western blot analysis of cCASP3/CASP3 **(I)** and cPARP/PARP **(J)** expression in PBMCs, isolated from *TP53*β WT and *TP53*β N340D control individuals and breast cancer patients, following 1 µM DXR treatment for 72 h. Data are presented as mean ± SEM (*N* = 2–8). * *p* < 0.05; *** *p* < 0.001. **(K)** Representative western blot images of DNA damage and apoptotic markers in PBMCs. PBMCs: peripheral blood mononuclear cells; *TP53*: tumour suppressor protein 53; WT: wild type; DXR: doxorubicin; C: control; BC: breast cancer; PI: propidium iodide.

The functional impact of the *TP53*β N340D variant on PBMC proliferation was assessed following LPS and PHA-L stimulation. All LPS-treated groups showed a significant increase in cell proliferation compared to untreated controls, which comprised untreated PBMCs from all participants (99.1, 87.4–112, *p* < 0.001) (Fig. 2A). Compared to *TP53*β WT controls (352, 277–428), both *TP53*β WT (236, 169–332, *p* = 0.0237) and *TP53*β N340D (222, 161–290, *p* = 0.0359) breast cancer groups exhibited significantly lower cell proliferation following LPS treatment (Fig. 2A). Similarly, all PHA-L-treated groups indicated a significant increase in cell proliferation compared to untreated controls (99.1, 87.4–112, *p* < 0.001) (Fig. 2B). Following PHA-L stimulation, *TP53*β WT (157, 111–194, *p* < 0.001) and *TP53*β N340D (177, 119–243, *p* = 0.0142) breast cancer groups showed significantly lower cell proliferation compared to *TP53*β WT controls (258, 212–312) (Fig. 2B).

Likewise, the variant’s impact on PBMC death after chemotherapy was evaluated. Comparisons to untreated controls (99.1, 87.4–112) revealed that all groups showed a significant decrease (*p* < 0.001) in mitochondrial reductive capacity (increased cell death) after DXR treatment, except for the *TP53*β N340D breast cancer group (103, 74.5–132, *p* > 0.05) (Fig. 2C). When comparing DXR-treated groups, the *TP53*β WT breast cancer group (64.1, 50.4–81.2) exhibited significantly higher mitochondrial reductive capacity (decreased cell death) than *TP53*β WT controls (43.4, 29.2–63.3, *p* = 0.0193) (Fig. 2C). The *TP53*β N340D breast cancer group (103, 74.5–132) showed significantly higher mitochondrial reductive capacity (decreased cell death) compared to *TP53*β WT controls (43.4, 29.2–63.3), *TP53*β WT breast cancer (64.1, 50.4–81.2), and *TP53*β N340D controls (60.1, 34.9–81.7) (*p* < 0.001) (Fig. 2C). All 5-FU, etoposide, and GEM-treated groups indicated a significant decrease in mitochondrial reductive capacity (increased cell death) compared to untreated controls (*p* < 0.001) (Fig. 2D-F). However, no significant differences were observed between the *TP53* groups (*p* > 0.05) (Fig. 2D-F). Based on these results, DXR treatment was used for downstream analyses.

#### Flow cytometry assay

An Annexin V and PI assay revealed significantly more healthy cells in the *TP53*β N340D breast cancer group (−46.2 ± 9.57) compared to *TP53*β WT controls (−58.8 ± 2.57) following DXR treatment (*p* = 0.0421) (Fig. 2G-H). Furthermore, significantly lower levels of late apoptosis were observed in the *TP53*β N340D breast cancer group (31.8 ± 9.50) compared to *TP53*β WT controls (48.6 ± 3.07) after DXR treatment (*p* = 0.00351) (Fig. 2G-H).

#### Western blotting

Apoptotic signalling responses in PBMCs were assessed following DXR treatment. A significant increase in cCASP3/CASP3 expression was detected in the DXR-treated *TP53*β WT group (180 ± 34.6) compared to its untreated group (72.5 ± 21.0) (*p* = 0.0404) (Fig. 2I). In contrast, no significant differences were observed between the DXR-treated (264 ± 41.4) and untreated (182 ± 46.2) *TP53*β N340D groups (*p* > 0.05) (Fig. 2I). As with cCASP3/CASP3, c-PARP/PARP expression was significantly increased in the DXR-treated *TP53*β WT group (734 ± 91.4) compared to its untreated counterpart (113 ± 10.9) (*p* < 0.001) (Fig. 2J). Moreover, no significant differences were observed among the DXR-treated (298 ± 48.7) and untreated (64.0 ± 5.27) *TP53*β N340D groups (*p* = 0.167) (Fig. 2J). Additionally, c-PARP/PARP expression in the DXR-treated *TP53*β N340D group (298 ± 48.7) was significantly lower than in the DXR-treated *TP53*β WT group (734 ± 91.4) (*p* < 0.001) (Fig. 2J). No significant differences were observed in the expression of pH2AX/H2AX, PUMA, and BAX between the groups (*p* > 0.05) (Fig. 2K, Figure A.11).

### Cellular senescence

#### Cell morphology

**Fig. 3:**
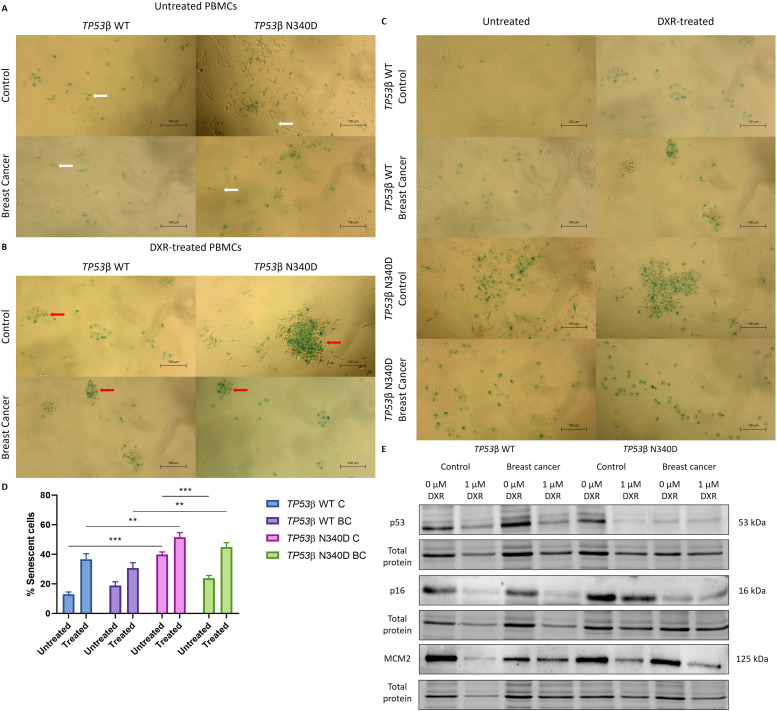
Morphological assessment of untreated **(A)** and DXR-treated **(B)** PBMCs, isolated from *TP53*β WT and *TP53*β N340D control individuals and breast cancer patients. White arrow – Elongated spindle-shaped cells. Red arrow – “Colony”-forming senescent cells. **(C)** Representative images of SA β-Gal-stained PBMCs. **(D)** Percentage of SA β-Gal positive cells in untreated and DXR-treated PBMCs, isolated from *TP53*β WT and *TP53*β N340D control individuals and breast cancer patients. Data are presented as mean ± SEM (*N* = 2). ** *p* < 0.01; *** *p* < 0.001. **(E)** Representative western blot images of senescence and DNA replication markers in PBMCs, isolated from *TP53*β WT and *TP53*β N340D control individuals and breast cancer patients, following 1 µM DXR treatment for 72 h. PBMCs: peripheral blood mononuclear cells; *TP53*: tumour suppressor protein 53; WT: wild type; DXR: doxorubicin; C: control; BC: breast cancer; SA β-Gal: senescence-associated beta-galactosidase.

In the absence of DXR treatment, more elongated, spindle-shaped PBMCs were observed in the *TP53*β N340D control group compared to the other groups. (Fig. 3A). Furthermore, more “colony”-forming senescent PBMCs were observed following DXR treatment in this group compared to the other groups (Fig. 3B).

#### Senescence-associated beta-galactosidase assay

Overall, all DXR-treated groups exhibited significantly higher senescence than their untreated controls (Fig. 3C-D). Untreated *TP53*β N340D controls (307 ± 13.6) showed a significantly higher senescence compared to untreated *TP53*β WT controls (100 ± 11.6) (*p* < 0.001) (Fig. 3C-D). Furthermore, a significantly higher senescence was observed in DXR-treated *TP53*β N340D controls (396 ± 24.4) compared to DXR-treated *TP53*β WT controls (282 ± 29.2) (*p* = 0.00250) (Fig. 3C-D). Similarly, a significantly higher senescence was observed in the DXR-treated *TP53*β N340D breast cancer group (346 ± 22.9) compared to the DXR-treated *TP53*β WT breast cancer group (237 ± 27.6) (*p* = 0.00488) (Fig. 3C-D). The untreated *TP53*β N340D breast cancer group (183 ± 14.9) exhibited a significantly reduced senescence compared to untreated *TP53*β N340D controls (307 ± 13.6) (*p* < 0.001) (Fig. 3C-D).

#### Western blotting

Western blot analysis was performed to assess the effect of the *TP53*β N340D variant on the expression of senescence and DNA replication markers in PBMCs following DXR treatment. No significant differences in the expression levels of p53, p16 or MCM2 were observed among the groups (*p* > 0.05) (Fig. 3E, Figure A.12).

### Cell migration

**Fig. 4:**
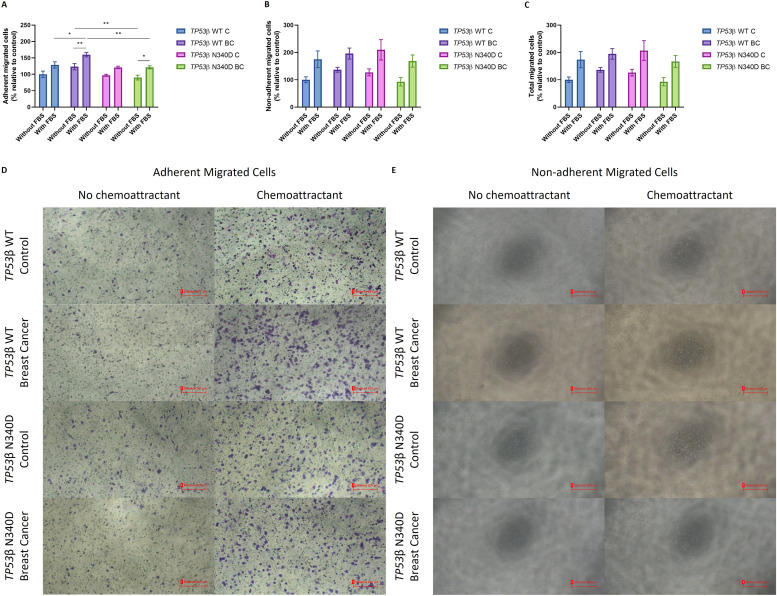
Migratory capacity of PBMCs isolated from *TP53*β WT and *TP53*β N340D control individuals and breast cancer patients, in the absence or presence of the chemoattractant (10 % HI-FBS), after 24 h. Data are presented as mean ± SEM (*N* = 2). **(A)** Number of adherent migrated PBMCs. * *p* < 0.05; ** *p* < 0.01. **(B)** Number of non-adherent migrated PBMCs. **(C)** Total number of migrated PBMCs. **(D)** Representative images of crystal violet-stained adherent PBMCs on the underside of the transwell insert. **(E)** Representative images of non-adherent PBMCs at the bottom of the well that did not adhere to the transwell insert. PBMC: peripheral blood mononuclear cells; *TP53*: tumour suppressor protein 53; WT: wild type; C: control; BC: breast cancer; HI-FBS: heat-inactivated fetal bovine serum.

A Transwell assay revealed a significant increase in adherent PBMC migration in the *TP53*β WT breast cancer group (160 ± 6.57) compared to *TP53*β WT controls (129 ± 9.40) (*p* = 0.0384) (Fig. 4A, 4D). Migration was significantly reduced in the *TP53*β N340D breast cancer group (121 ± 4.73) compared to the *TP53*β WT breast cancer group (160 ± 6.57) (*p* = 0.00441) (Fig. 4A, 4D). In contrast to adherent PBMCs, no significant differences in non-adherent PBMC migration were observed across the groups (Fig. 4B, 4E). Furthermore, the total number of migrated PBMCs, combining adherent and non-adherent cell counts, indicated no significant differences between the groups (Fig. 4C).

#### PBMC-mediated anti-tumour activity

**Fig. 5:**
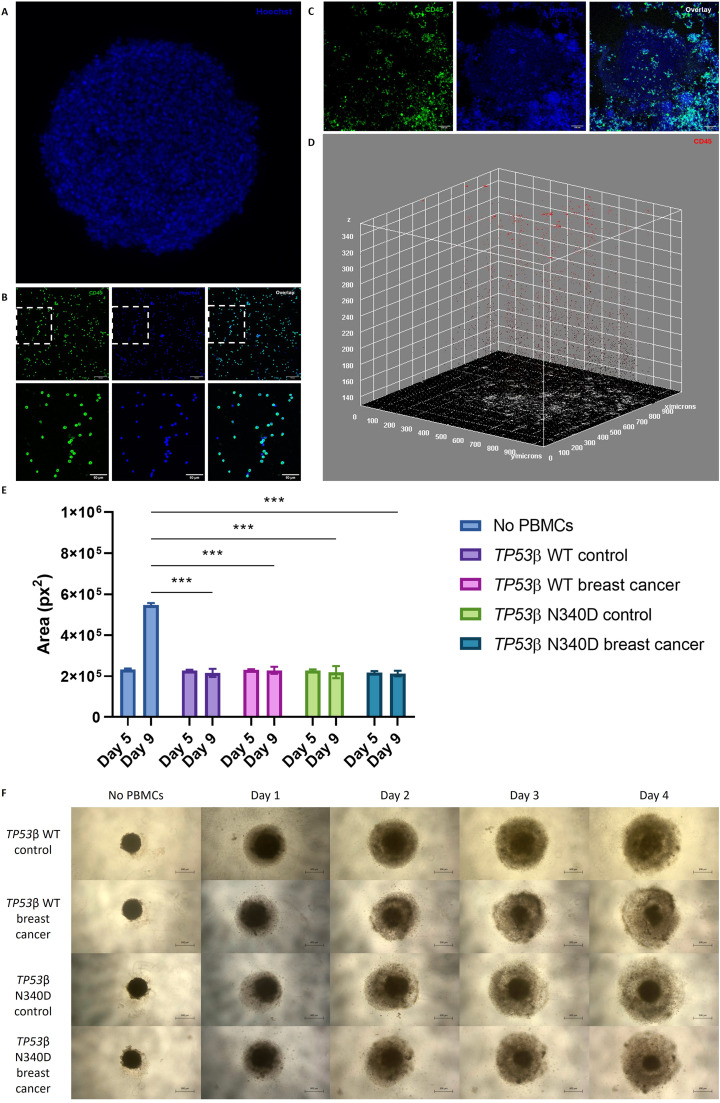
Co-culturing of BT-549 breast cancer spheroid and PBMCs, stained with CD45 marker (Alexa Fluor 488) and Hoechst. **(A)** BT-549 spheroid, 10X objective. **(B)** PBMCs, 20X objective. **(C)** PBMC co-culture with BT-549 spheroids, 10X objective. **(D)** 3D surface plot of PBMC distribution following co-culture with BT-549 spheroids in a Nunclon™ Sphera™ 96-well U-bottom well (exclusion of BT-549 spheroid), 10X objective. **(E)** PBMC-mediated destruction of BT-549 spheroids, measured by spheroid size, following four days of co-culture with PBMCs from *TP53*β WT and *TP53*β N340D control individuals and breast cancer patients. Data are presented as mean ± SEM (*N* = 2–3). *** *p* < 0.001. **(F)** Representative images of spheroid. PBMCs: peripheral blood mononuclear cells; *TP53*: tumour suppressor protein 53; WT: wild type.

To determine the impact of the *TP53*β N340D variant on PBMC-mediated anti-tumour activity, a 3D co-culture spheroid model was established (Fig. 5A-D). Morphological assessment indicated reduced spheroid disruption after co-culturing with *TP53*β N340D PBMCs compared to *TP53*β WT PBMCs (Fig. 5F). There were no significant differences in spheroid size among the groups (Fig. 5E). All groups showed a significant decrease in spheroid size compared to spheres without PBMC co-culture (*p* < 0.001) (Fig. 5E).

## Discussion

This study investigated the hypothesis that the germline *TP53*β N340D variant compromises key functional responses in PBMCs, affecting both healthy individuals and breast cancer patients, compared to *TP53*β WT counterparts.

### TP53β N340D variant impairs apoptotic response in PBMCs from breast cancer patients

p53 is a central mediator of the DNA damage response, guiding cell fate outcomes such as cell cycle arrest, DNA repair, senescence, and apoptosis following genotoxic stress [13]. In this study, we demonstrate that the *TP53*β N340D variant may affect these processes. PBMCs from breast cancer patients carrying the *TP53*β N340D variant exhibited increased resistance to DXR-induced cytotoxicity (Fig. 2C). No differences were observed between *TP53*β N340D controls and *TP53*β WT controls, suggesting that additional cancer-specific genetic or environmental factors may exacerbate the variant’s functional impact (Fig. 2C). These findings point to a compromised cell death response associated with the *TP53*β N340D variant in the context of breast cancer. This is consistent with flow cytometry and western blot data (Figs. 2H and 2I-J), which showed reduced late apoptosis and a failure to activate key apoptotic proteins, such as CASP3 and PARP, in *TP53*β N340D PBMCs in response to DXR. As such, the *TP53*β N340D variant may promote cell survival under genotoxic stress. Downstream analysis of direct p53 transcriptional targets, including BAX and PUMA, revealed no significant differences between groups (Fig. 2K, Figure A.11). However, the expression and activity these markers are highly dynamic and time dependent, therefore assessing them at a single time point (e.g., 72 h) may have overlooked transient transcriptional changes. Furthermore, the impaired apoptotic response may be mediated through alternative downstream p53 effectors or regulatory mechanisms influenced by the *TP53*β N340D variant.

### TP53β N340D variant contributes to cellular senescence in PBMCs

Senescent cells undergo distinct morphological and structural changes [14]. In this study, untreated PBMCs from *TP53*β N340D controls exhibited a more elongated, spindle-shaped morphology, whereas their DXR-treated PBMCs displayed increased "colony"-like formations (Fig. 3A-B). Similar morphological features have previously been reported in PBMCs following irradiation [15] and are consistent with cytoskeletal remodelling processes linked to p53 dysregulation [16].

Furthermore, senescent cells exhibit increased lysosomal activity, reflected by elevated SA β-gal staining [14]. In this study, PBMCs from *TP53*β N340D controls showed increased basal senescence compared to *TP53*β WT controls (Fig. 3C-D), even in the absence of exogenously induced stress, suggesting an endogenous imbalance for senescence induction to occur. This heightened senescence could represent a compensatory tumour-suppressive response to potentially compromised cells, aiming to limit its proliferation [2,3]. The *TP53*β N340D breast cancer patients exhibited significantly lower basal senescence than their non-cancer counterparts (Fig. 3C-D), reflecting an impaired activation of this protective response.

Following DXR treatment, both *TP53*β N340D control and breast cancer groups exhibited increased senescence compared to *TP53*β WT counterparts, suggesting a shift from apoptosis to senescence as the cell fate outcome (Fig. 3C-D), potentially promoting chemoresistance and cancer recurrence through senescence-associated secretory phenotype (SASP) signalling. Molecular analysis of senescence and DNA replication markers, including p53, p16, and MCM2 [17,18], revealed no significant differences across the groups (Fig. 3E, Figure A.12), suggesting that the senescent response may be mediated by altered post-translational modifications, such as p53 or p16 phosphorylation, or alternative signalling pathways, such as p21 activation, influenced by the *TP53*β N340D variant.

The increased senescence observed in *TP53*β N340D PBMCs may arise from the unique regulatory functions of the p53β isoform, which differ from p53α-mediated tumour suppression. Unlike full-length (FL) p53α, the p53β isoform lacks the oligomerization domain, the region essential for tetramerization, and possesses a distinct C-terminal region that enhances transcriptional activation of senescence markers [19]. Previous studies have shown that overexpression of p53β induces cellular senescence, whereas *TP53*β knockout reduces markers of cellular senescence, particularly under conditions of DNA damage [20]. The N340D variant occurs within the C-terminal domain of p53β, potentially shifting the transcriptional balance toward senescence. Importantly, this contrasts with conventional *TP53* loss-of-function mutations that typically abolish DNA-binding activity, leading to uncontrolled proliferation or genomic instability [21].

### TP53β N340D variant does not affect cell proliferation in PBMCs

Monocytes and T lymphocytes can be stimulated by LPS and PHA-L, respectively, to induce cell proliferation [22]. Cancer patients often present a compromised immune system, characterised by reduced immune cell proliferation [23], as observed in both breast cancer groups compared to *TP53*β WT controls in our study (Fig. 2A-B). No significant differences were found between the *TP53*β WT and *TP53*β N340D groups within both control and breast cancer cohorts, suggesting that breast cancer itself, rather than the *TP53*β variant, was the primary factor influencing proliferation (Fig. 2A-B).

### TP53β N340D variant reduces cell migration in adherent PBMCs in the context of cancer

p53 regulates cell migration and invasion through processes such as filopodia formation, cellular polarization and spreading [24]. Immune cell migration is essential for effective immune surveillance and recruitment, enabling the elimination of cancerous cells and thereby restricting tumour progression [25]. In this study, adherent PBMCs, primarily comprising monocytes and dendritic cells, from *TP53*β N340D breast cancer patients exhibited a significantly impaired migratory capacity compared to *TP53*β WT breast cancer patients (Fig. 4A, 4D). This reduced migratory ability may compromise immune cell infiltration and tumour cell targeting, potentially contributing to immune evasion in these patients.

### TP53β N340D variant does not affect PBMC-mediated anti-tumour activity

Morphological assessment of spheroid integrity showed reduced spheroid disruption in *TP53*β N340D PBMC groups (Fig. 5F). However, no statistically significant differences in spheroid size could be found (Fig. 5E). As such, further investigation is required to explore the potential impact of the *TP53*β N340D variant on PBMC-mediated anti-tumour activity.

### Relationship between cellular phenotypes and clinical characteristics

Germline *TP53* mutations have been associated with more aggressive tumours, therapeutic resistance, and a poor prognosis [26]. Additionally, the age of cancer onset is reportedly linked to the functional severity of the *TP53* mutation, which confers varying degrees of penetrance [27]. The cellular phenotypes associated with the *TP53*β N340D variant provide mechanistic insight into the clinical presentation observed in the affected family. The index patient developed multiple primary breast carcinomas between the ages of 30 and 60 years, consistent with a Li-Fraumeni-like phenotype that often arises from germline *TP53* mutations [10]. The impaired apoptotic response observed in *TP53*β N340D PBMCs, together with enhanced cellular senescence, suggests that this variant may promote the survival of genomically compromised cells while simultaneously driving premature immune exhaustion. This dual defect could accelerate age-related immune decline and reduce the ability to eliminate emerging tumour cells, contributing to early disease onset.

In addition, the shift from apoptosis to senescence in response to genotoxic stress, as seen following doxorubicin exposure, may explain suboptimal treatment responses and cancer recurrence following radiotherapy in the index patient [10]. This aligns with previous reports that persistent senescent cells can create a pro-inflammatory microenvironment through SASP activation, fostering secondary malignancies and tissue damage following therapy [28]. Therefore, the variant’s functional impairments may underlie both the penetrance and treatment-related complications observed clinically, reinforcing the need for precision oncology approaches when managing carriers of germline *TP53*β variants.

## Conclusion

**Fig. 6:**
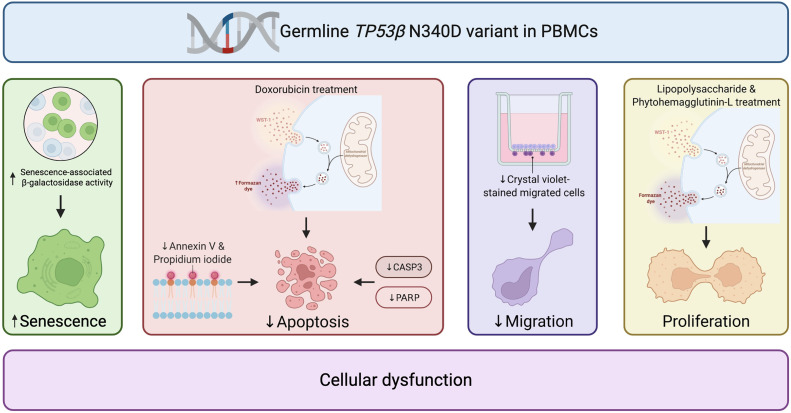
The germline *TP53*β N340D variant enhances cellular senescence, impairs apoptotic responses, and reduces cell migration in PBMCs, with no effect on cell proliferation, collectively contributing to cellular dysfunction in breast cancer patients. PBMCs: peripheral blood mononuclear cells; *TP53*: tumour suppressor protein 53.

This study highlights the importance of integrating functional genomics into the PSGT framework when rare novel variants or VUS are identified. Moreover, these findings support the hypothesis that the germline *TP53*β N340D variant compromises key functional responses in PBMCs, including increased cellular senescence, impaired apoptosis, and reduced cell migration (Fig. 6). The cellular phenotypes associated with the *TP53*β N340D variant may explain the disease penetrance, early-onset cancer, and cancer recurrence following radiotherapy, highlighting the critical role of the three-pronged PSGT approach that integrates inherited, lifestyle and therapy-related factors to guide variant interpretation and clinical decision-making.

It is important to acknowledge the small sample size, particularly in the *TP53*β N340D groups, due to the low population frequency of the variant. Since each participant's PBMCs reflect not only their genetic makeup but also physiological and environmental factors, this inherent variability may have introduced confounding variables. Advances in genetic engineering may soon enable CRISPR editing at the *TP53*β N340D site in induced pluripotent stem cells. These edited cells could be differentiated into breast epithelial cells to model disease more accurately. Such models would allow analysis of p53β expression and localisation (using isoform-specific antibodies like KJC8 [29]), as well as interaction networks, revealing the variant’s broader cellular effects.

In line with the American College of Medical Genetics and Genomics and Association for Molecular Pathology guidelines, this study provides supporting functional evidence for the pathogenicity of the *TP53*β N340D variant. Beyond ClinGen’s *TP53* Expert Panel specifications, the integration of clinical data extending beyond the index case’s family history strengthens the rationale for returning research results as part of the envisaged Open Genome Project, which aims to eliminate cost barriers to the implementation of personalized medicine.

## Ethics approval and consent to participate

This study was performed in line with the principles of the World Medical Association Declaration of Helsinki. Ethics approval was granted by the Health Research Ethics Committee of Stellenbosch University for the “Application of personalized medicine using an integrated service and research approach: The Open Genome Project” (Project ID: 10,648; Reference number: C19/06/020). Informed consent and completed case report forms were obtained from all participants included in the study.

## Consent for publication

The authors affirm that human research participants provided informed consent for publication.

## Data availability

The datasets generated during the current study are available from the corresponding author on reasonable request, with the experimental methods described in detail in the PhD dissertation of the first author [10].

## Funding sources

This work was funded by the National Research Foundation (Grant number: 138118). Support from the South African Medical Research Council (SAMRC) included funding received from the Department of Science and Innovation (Grant number: 96852). The South African BioDesign Initiative of the Department of Science and Innovation and the Technology Innovation Agency are acknowledged for funding part of this research under the Open Genome Project (Grant number: 401/01). Any opinions, findings, conclusions or recommendations expressed in this article are those of the authors, and the funders accept no liability in this regard.

## CRediT authorship contribution statement

**Claudia Christowitz:** Writing – review & editing, Writing – original draft, Methodology, Investigation, Formal analysis, Data curation, Conceptualization. **Daniel W Olivier:** Writing – review & editing, Methodology, Conceptualization. **Nicole van der Merwe:** Writing – review & editing, Project administration, Conceptualization. **Maritha J Kotze:** Writing – review & editing, Project administration, Funding acquisition, Conceptualization. **Anna-Mart Engelbrecht:** Writing – review & editing, Supervision, Resources, Funding acquisition, Conceptualization.

## Declaration of competing interest

The authors declare the following which may be considered as potential competing interests: MJ Kotze is a founder director and shareholder of Gknowmix Pty Ltd., to facilitate genetic research translation into clinical practice. DW Olivier is a consultant for Gknowmix Pty Ltd. The other authors have no conflict interest to declare.

## Appendix Supplementary materials

Image, application 1

## References

[1] Bray F., Laversanne M., Sung H. (2024). Global cancer statistics 2022: GLOBOCAN estimates of incidence and mortality worldwide for 36 cancers in 185 countries. CA Cancer J. Clin..

[2] Fouad Y.A., Aanei C. (2017). Revisiting the hallmarks of cancer. Am. J. Cancer Res..

[3] Hanahan D. (2022). Hallmarks of cancer: new dimensions. Cancer Discov..

[4] Kotze M.J., Grant K.A., van der Merwe N.C., Barsdorf N.W., Kruger M. (2024). Navigating ethical challenges of integrating genomic medicine into clinical practice: maximising beneficence in precision oncology. South Afr. J. Bioeth. Law.

[5] Okunola A.O., Baatjes K.J., Zemlin A.E. (2023). Pathology-supported genetic testing for the application of breast cancer pharmacodiagnostics: family counselling, lifestyle adjustments and change of medication. Expert. Rev. Mol. Diagn..

[6] De Klerk M., Van Der Merwe N., Erasmus J. (2025). Incorporating familial risk, lifestyle factors, and pharmacogenomic insights into personalized noncommunicable disease (NCD) reports for healthcare funder beneficiaries participating in the open genome project. Ann. Hum. Genet..

[7] Christowitz C., Olivier D.W., Schneider J.W., Kotze M.J., Engelbrecht A.M. (2024). Incorporating functional genomics into the pathology-supported genetic testing framework implemented in South Africa: a future view of precision medicine for breast carcinomas. Mutat. Res. - Rev. Mutat. Res..

[8] Leroy B., Ballinger M.L., Baran-Marszak F. (2017). Recommended guidelines for validation, quality control, and reporting of TP53 variants in clinical practice. Cancer Res..

[9] Kotze M.J., Peeters A., Pienaar R., Baatjes K.J. (2019). Family screening and data sharing towards variant classification of TP53 c.1018A>G (N340D) that targets isoform beta. Breast.

[10] Christowitz C. (2025). The functional impact of a rare germline TP53 β N340D variant identified in breast cancer patients with the Li-Fraumeni-like syndrome. https://scholar.sun.ac.za/items/f4c7939a-8955-4a66-b047-ce2b1a72861c.

[11] Van der Merwe N.C., Ntaita K.S., Stofberg H., Combrink H.M.E., Oosthuizen J., Kotze M.J. (2022). Implementation of multigene panel testing for breast and ovarian cancer in South Africa: a step towards excellence in oncology for the public sector. Front. Oncol..

[12] Glanzmann B., Jooste T., Ghoor S. (2021). Human whole genome sequencing in South Africa. Sci. Rep..

[13] Steffens Reinhardt L., Groen K., Newton C., Avery-Kiejda K.A (2023). The role of truncated p53 isoforms in the DNA damage response. Biochim. Biophys. Acta - Rev. Cancer.

[14] Herranz N., Gil J. (2018). Mechanisms and functions of cellular senescence. J. Clin. Invest..

[15] Moonkum N., Wongpiem U., Mongkolsuk M., Pilapong C. Characteristic of peripheral blood mononuclear cells after diagnostic irradiation in term of morphology and differentiation potency. 2020; (May):412–418.

[16] Araki K., Ebata T., Guo A.K., Tobiume K., Wolf S.J., Kawauchi K. (2015). P53 regulates cytoskeleton remodeling to suppress tumor progression. Cell Mol. Life Sci..

[17] Sun Y., Cheng Z., Liu S. (2022). MCM2 in human cancer: functions, mechanisms, and clinical significance. Mol. Med..

[18] Yang J., Liu M., Hong D., Zeng M., Zhang X. (2021). The paradoxical role of cellular senescence in cancer. Front. Cell Dev. Biol..

[19] Ray Das S., Delahunt B., Lasham A. (2024). Combining TP53 mutation and isoform has the potential to improve clinical practice. Pathology..

[20] Chen J., Crutchley J., Zhang D., Owzar K., Kastan M.B. (2017). Identification of a DNA damage-induced alternative splicing pathway that regulates p53 and cellular senescence markers. Cancer Discov..

[21] Song B., Yang P., Zhang S. (2024). Cell fate regulation governed by p53: friends or reversible foes in cancer therapy. Cancer Commun..

[22] Lin Z., Huang Y., Jiang H. (2021). Functional differences and similarities in activated peripheral blood mononuclear cells by lipopolysaccharide or phytohemagglutinin stimulation between human and cynomolgus monkeys. Ann. Transl. Med..

[23] Bondhopadhyay B., Hussain S., Kasherwal V. (2023). The differential effect of the immune system in breast cancer. Explor. Med..

[24] Roger L., Gadea G., Roux P. (2006). Control of cell migration: a tumour suppressor function for p53?. Biol. Cell.

[25] Blagih J., Buck M.D., Vousden K.H. (2020). P53, Cancer and the Immune Response. J. Cell Sci..

[26] Blondeaux E., Arecco L., Punie K. (2023). Germline TP53 pathogenic variants and breast cancer: a narrative review. Cancer Treat. Rev..

[27] Amadou A., Waddington Achatz M.I., Hainaut P. (2018). Revisiting tumor patterns and penetrance in germline TP53 mutation carriers: temporal phases of Li-Fraumeni syndrome. Curr. Opin. Oncol..

[28] Ohtani N. (2022). The roles and mechanisms of senescence-associated secretory phenotype (SASP): can it be controlled by senolysis?. Inflamm. Regen..

[29] Bourdon J.-C., Fernandes K., Murray-Zmijewski F. (2005). p53 isoforms can regulate p53 transcriptional activity. Genes Dev..

