# Supplementary material for: First functional evidence that a rare germline TP53β variant drives senescence-associated immune suppression and impairs apoptosis and cell migration in breast cancer patients
Source: Transl Oncol. 2026 Jan 6;64:102616. doi: 10.1016/j.tranon.2025.102616 (PMC12813233; doi:10.1016/j.tranon.2025.102616)
Supplement: Supplementary file 1 [file mmc1.docx]

**Table A.1. Primary and secondary antibodies for Western blot analyses.**

| Primary antibody | Molecular weight (kDa) | Dilution | Secondary antibody | Dilution | Company (Catalogue number) |
| --- | --- | --- | --- | --- | --- |
| Western blotting | | | | | |
| p-H2AX | ~15 | 1:1000 | Anti-Rabbit (Cell Signalling Technology, 7074P2) | 1:10 000 | Cell Signalling Technology (9718S) |
| H2AX | ~15 | 1:1000 | Anti-Rabbit | 1:10 000 | Cell Signalling Technology (7631S) |
| PUMA | ~23 | 1:1000 | Anti-Rabbit | 1:10 000 | Cell Signalling Technology (12450T) |
| BAX | ~21 | 1:1000 | Anti-Rabbit | 1:10 000 | Cell Signalling Technology (5023T) |
| cCASP3 | ~17 | 1:1000 | Anti-Rabbit | 1:10 000 | Cell Signalling Technology (9661S) |
| CASP3 | ~32 | 1:1000 | Anti-Rabbit | 1:10 000 | Cell Signalling Technology (9662S) |
| cPARP, PARP | ~89, 116 | 1:1000 | Anti-Rabbit | 1:10 000 | Cell Signalling Technology (9532S) |
| p53 | ~53 | 1:1000 | Anti-Mouse (Cell Signalling Technology, 7076S) | 1:10 000 | Abcam (ab26) |
| p16 | ~16 | 1:1000 | Anti-Rabbit | 1:10 000 | Abcam (ab189034) |
| MCM2 | ~125 | 1:1000 | Anti-Rabbit | 1:10 000 | Abcam (ab108935) |
| Immunocytochemistry | | | | | |
| CD45 | N/A | 1:200 | Alexa Fluor® 488 Conjugate Anti-Rabbit (Thermo Fisher Scientific, A11001) | 1:200 | Thermo Fisher Scientific (14-9457-82) |


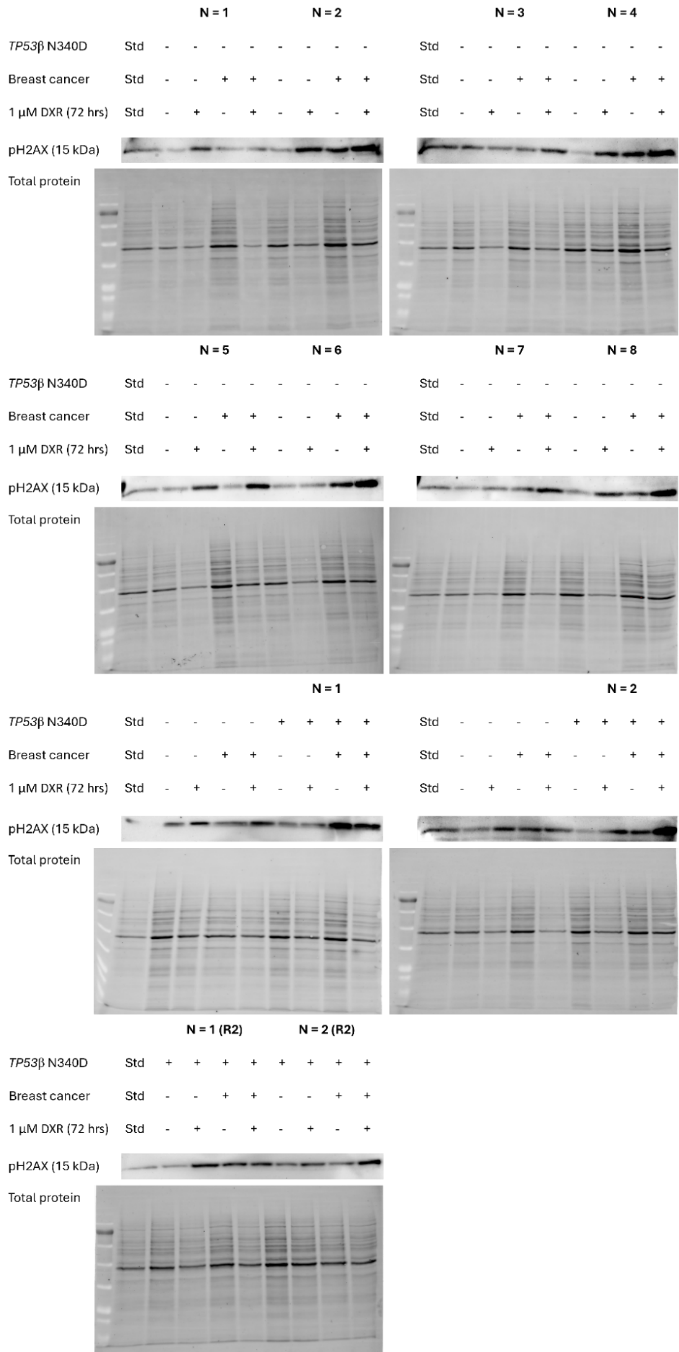


Figure A.1. Western blot images of p-H2AX expression in peripheral blood mononuclear cells, isolated from control individuals and breast cancer patients with or without the *TP53*β N340D variant, either untreated or treated with 1 µM DXR treatment for 72 hrs (N = 1-8). *TP53*: tumour suppressor protein 53; DXR: doxorubicin.


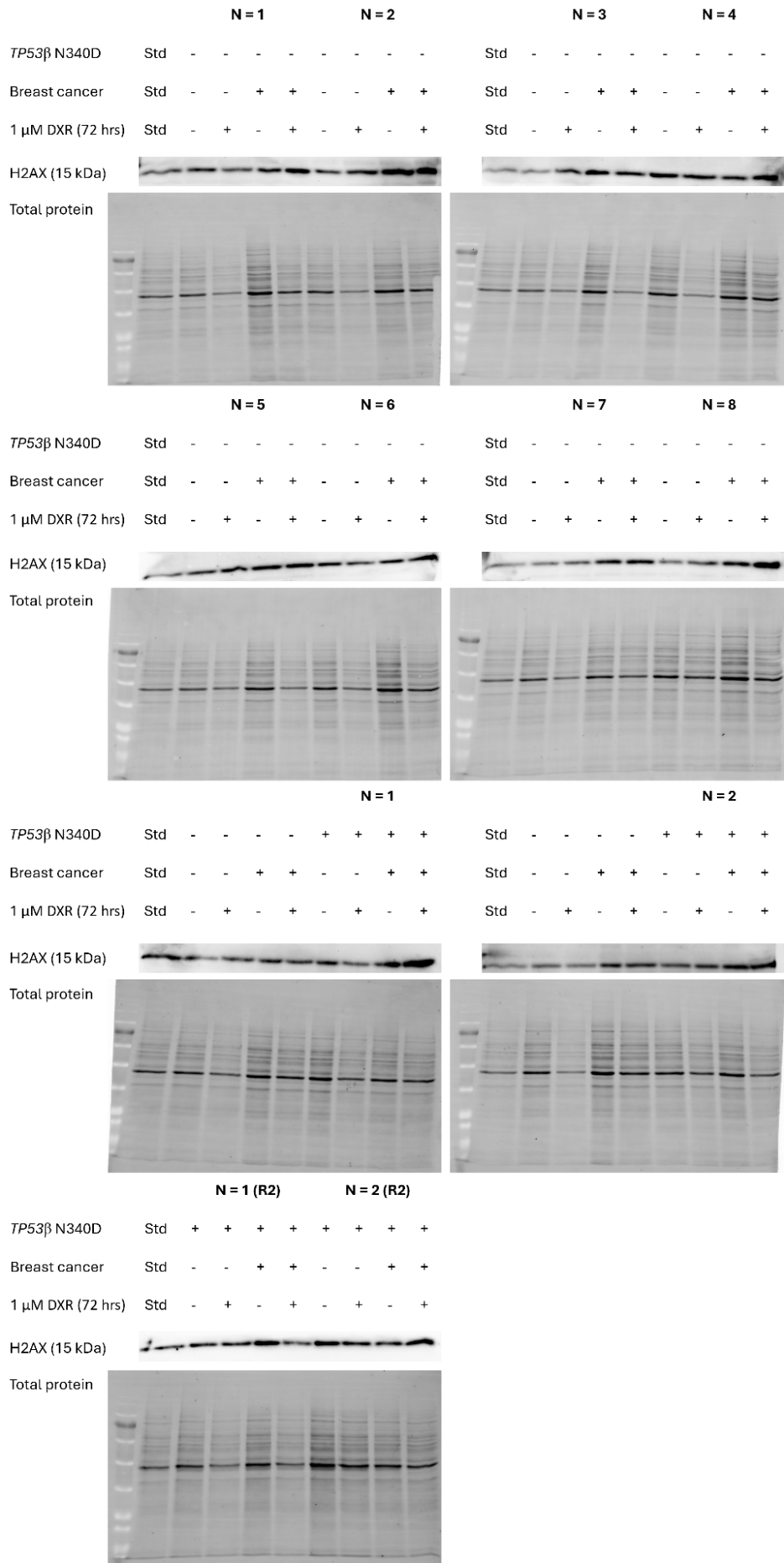


Figure A.2. Western blot images of H2AX expression in peripheral blood mononuclear cells, isolated from control individuals and breast cancer patients with or without the *TP53*β N340D variant, either untreated or treated with 1 µM DXR treatment for 72 hrs (N = 1-8). *TP53*: tumour suppressor protein 53; DXR: doxorubicin.


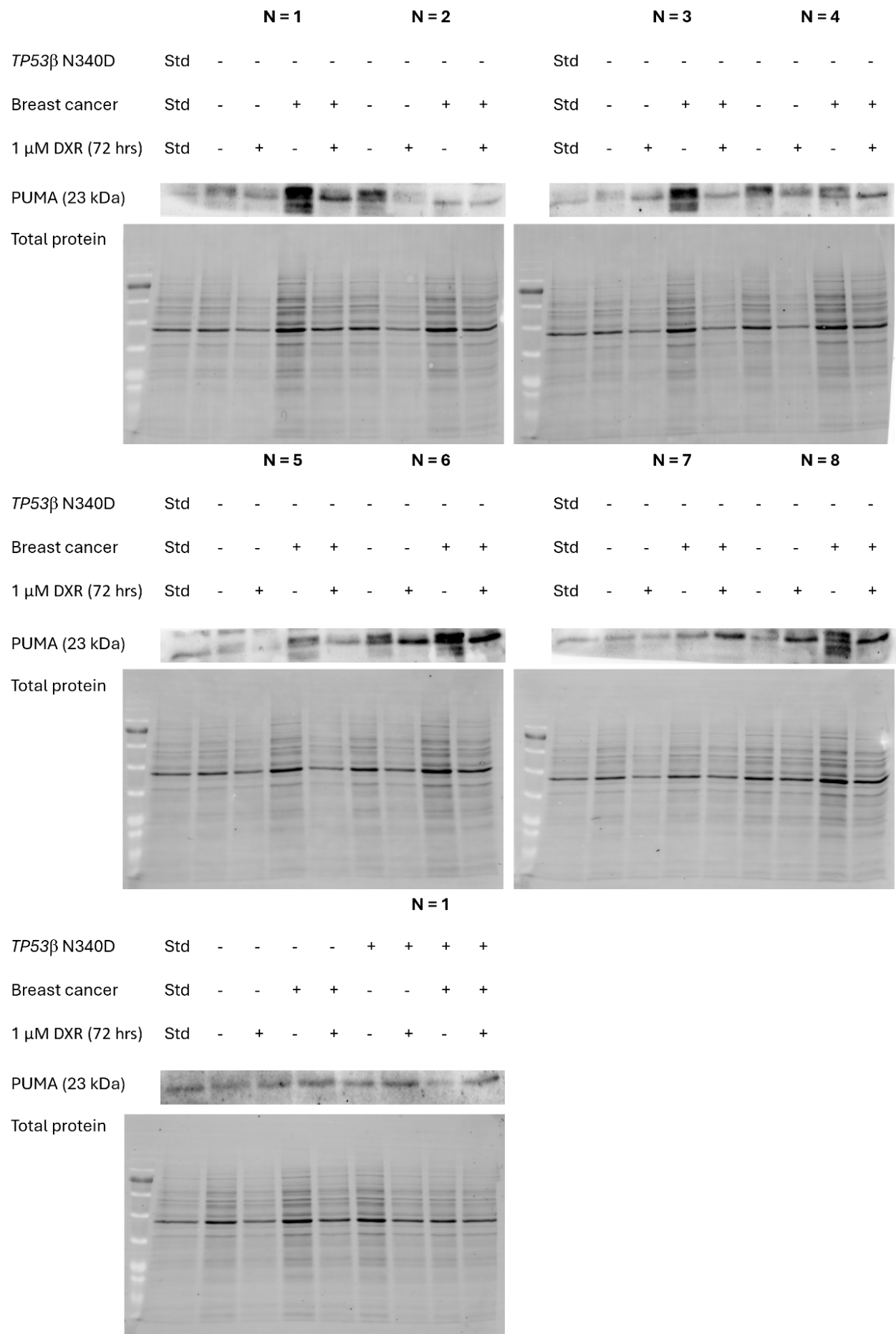


Figure A.3. Western blot images of PUMA expression in peripheral blood mononuclear cells, isolated from control individuals and breast cancer patients with or without the *TP53*β N340D variant, either untreated or treated with 1 µM DXR treatment for 72 hrs (N = 1-8). *TP53*: tumour suppressor protein 53; DXR: doxorubicin.


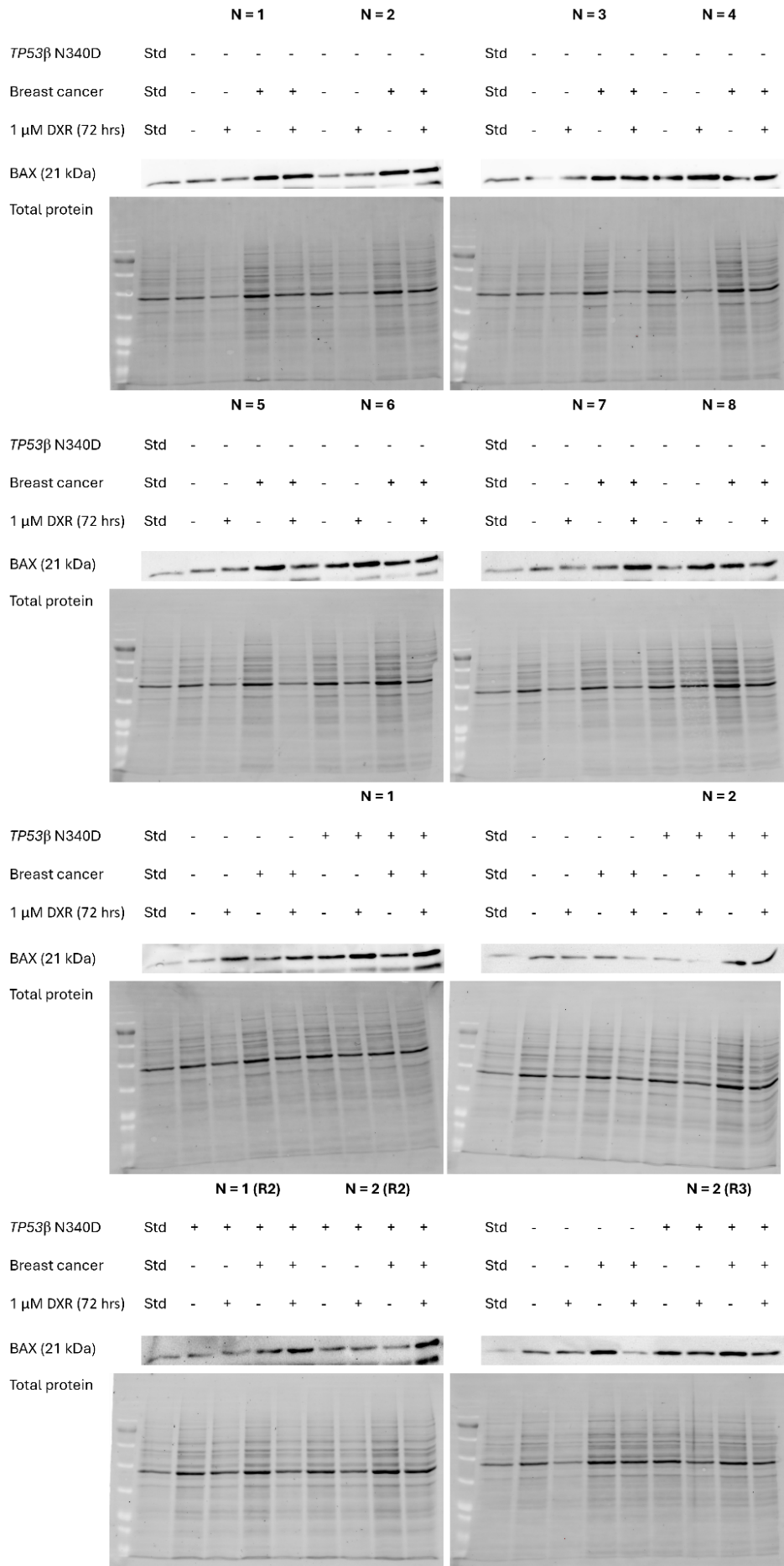


Figure A.4. Western blot images of BAX expression in peripheral blood mononuclear cells, isolated from control individuals and breast cancer patients with or without the *TP53*β N340D variant, either untreated or treated with 1 µM DXR treatment for 72 hrs (N = 1-8). *TP53*: tumour suppressor protein 53; DXR: doxorubicin.


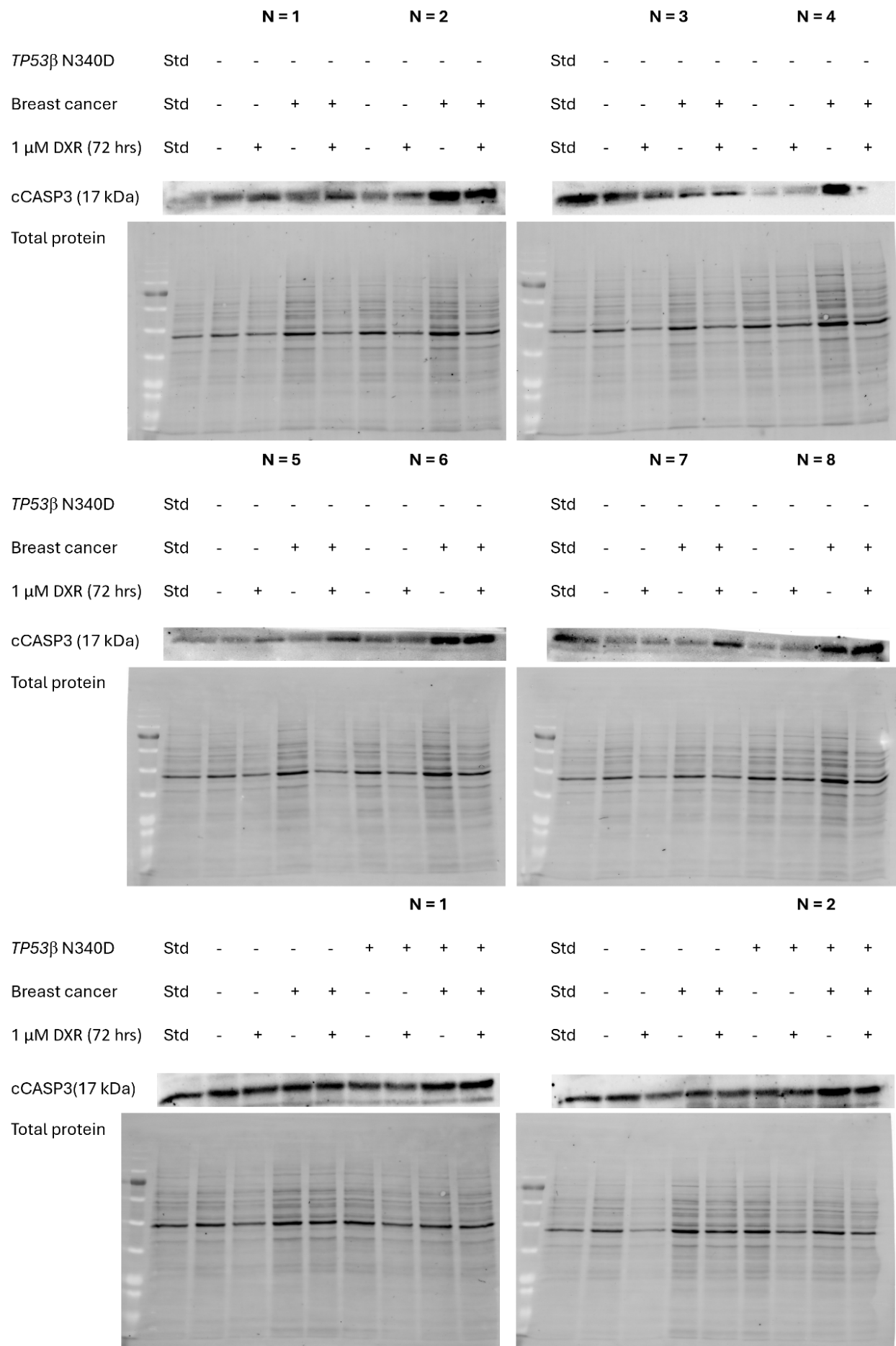


Figure A.5. Western blot images of cCASP3 expression in peripheral blood mononuclear cells, isolated from control individuals and breast cancer patients with or without the *TP53*β N340D variant, either untreated or treated with 1 µM DXR treatment for 72 hrs (N = 1-8). *TP53*: tumour suppressor protein 53; DXR: doxorubicin.


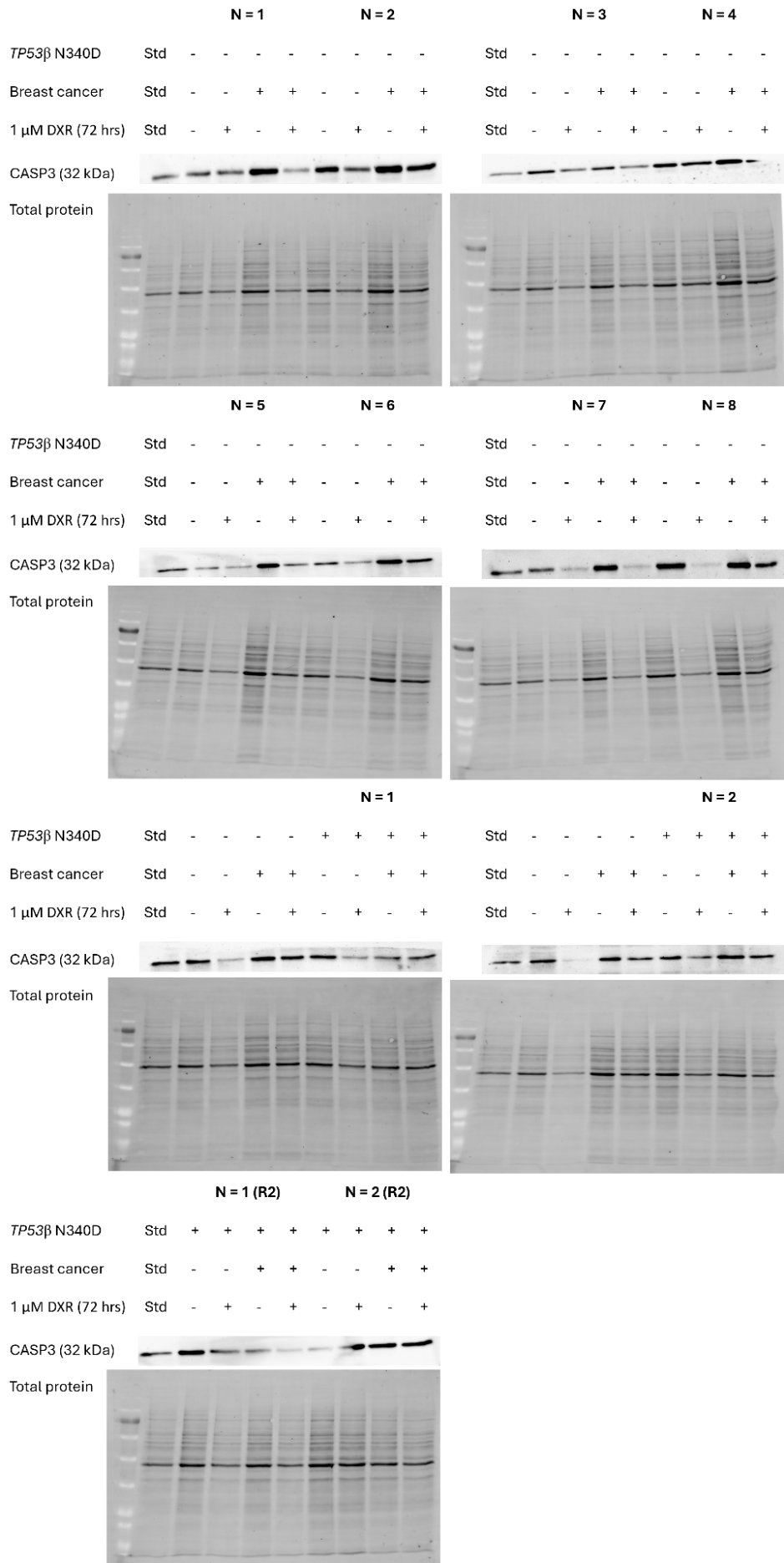


Figure A.6. Western blot images of CASP3 expression in peripheral blood mononuclear cells, isolated from control individuals and breast cancer patients with or without the *TP53*β N340D variant, either untreated or treated with 1 µM DXR treatment for 72 hrs (N = 1-8). *TP53*: tumour suppressor protein 53; DXR: doxorubicin.


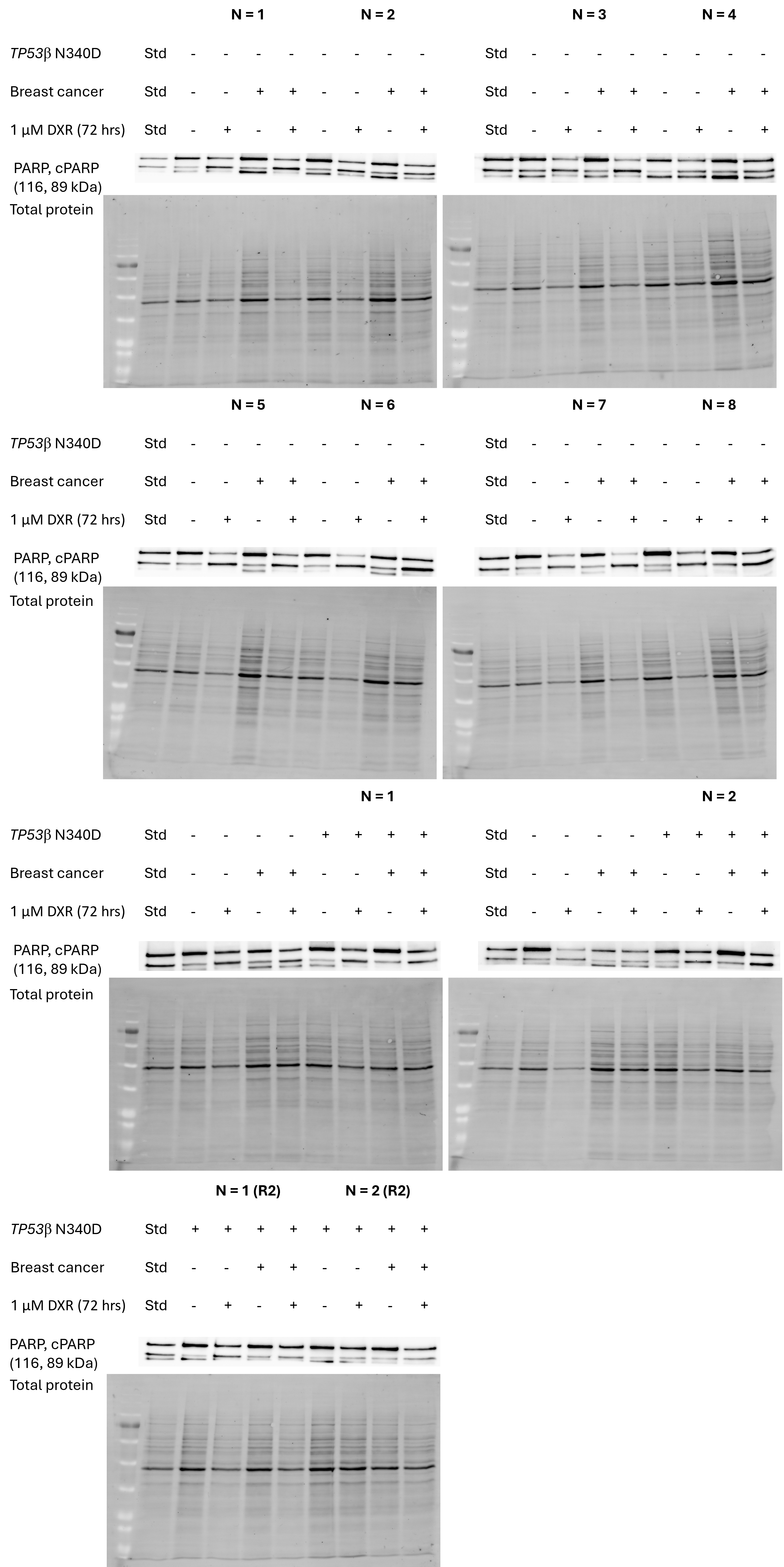


Figure A.7. Western blot images of cPARP and PARP expressions in peripheral blood mononuclear cells, isolated from control individuals and breast cancer patients with or without the *TP53*β N340D variant, either untreated or treated with 1 µM DXR treatment for 72 hrs (N = 1-8). *TP53*: tumour suppressor protein 53; DXR: doxorubicin.


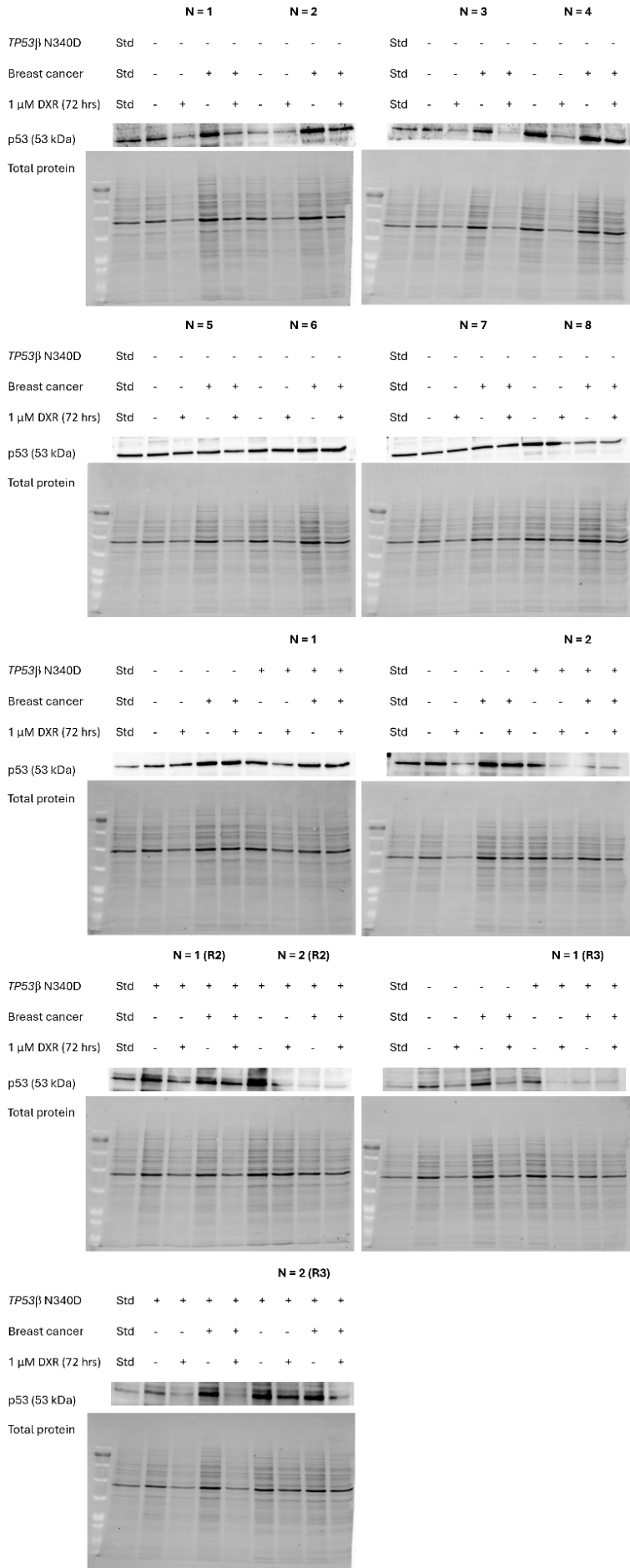


Figure A.8. Western blot images of p53 (FLp53α isoform) expression in peripheral blood mononuclear cells, isolated from control individuals and breast cancer patients with or without the *TP53*β N340D variant, either untreated or treated with 1 µM DXR treatment for 72 hrs (N = 1-8). *TP53*: tumour suppressor protein 53; DXR: doxorubicin.


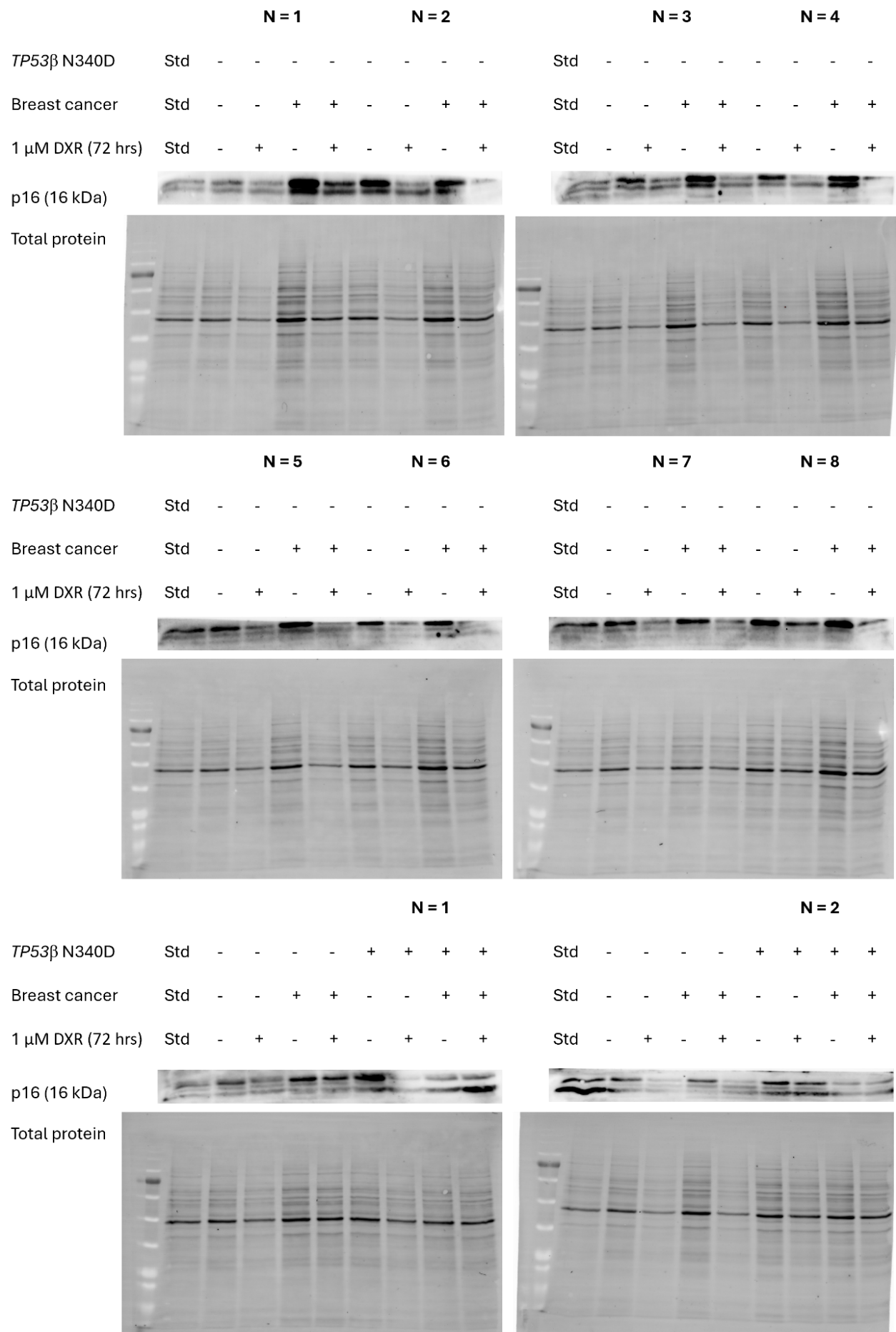


Figure A.9. Western blot images of p16 expression in peripheral blood mononuclear cells, isolated from control individuals and breast cancer patients with or without the *TP53*β N340D variant, either untreated or treated with 1 µM DXR treatment for 72 hrs (N = 1-8). *TP53*: tumour suppressor protein 53; DXR: doxorubicin.


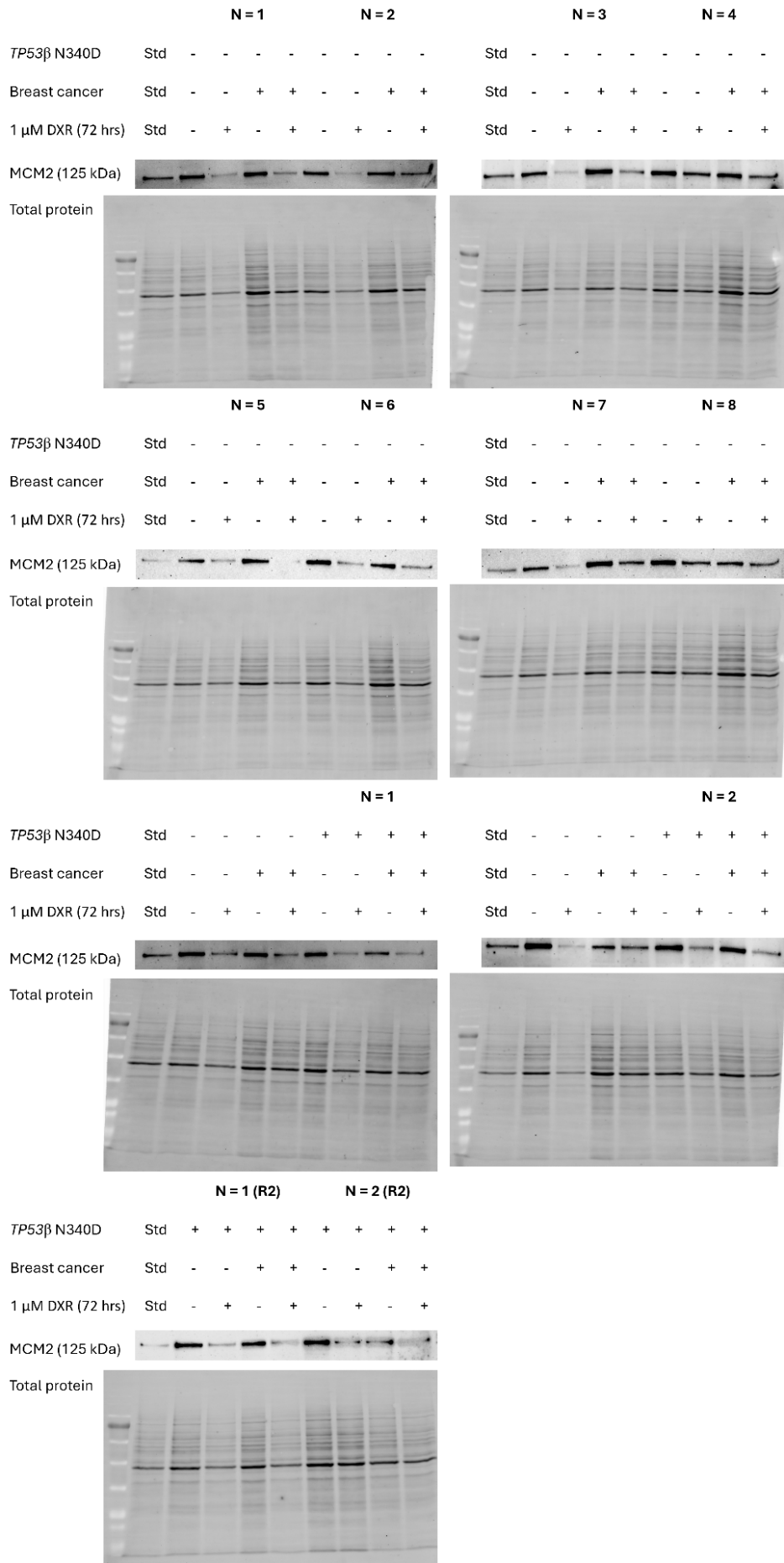


Figure A.10. Western blot images of MCM2 expression in peripheral blood mononuclear cells, isolated from control individuals and breast cancer patients with or without the *TP53*β N340D variant, either untreated or treated with 1 µM DXR treatment for 72 hrs (N = 1-8). *TP53*: tumour suppressor protein 53; DXR: doxorubicin.


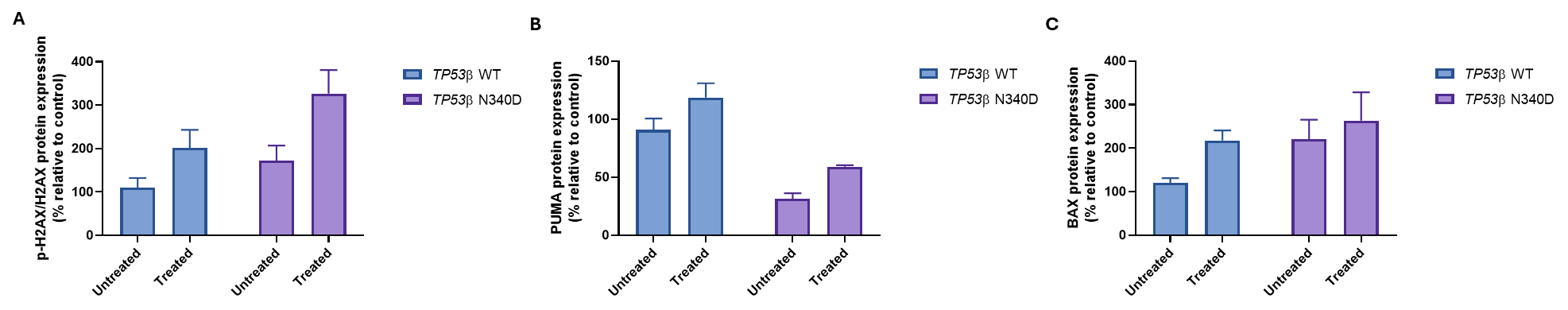


**Figure A.11.** Western blot analysis of p-H2AX/H2AX **(A)**, PUMA **(B)**, and BAX **(C)** expression in PBMCs, isolated from *TP53*β WT and *TP53*β N340D control individuals and breast cancer patients, following 1 µM DXR treatment for 72 hours. Data are presented as mean ± SEM (N = 2-8). PBMCs: peripheral blood mononuclear cells; *TP53*: tumour suppressor protein 53; WT: wild type; DXR: doxorubicin.


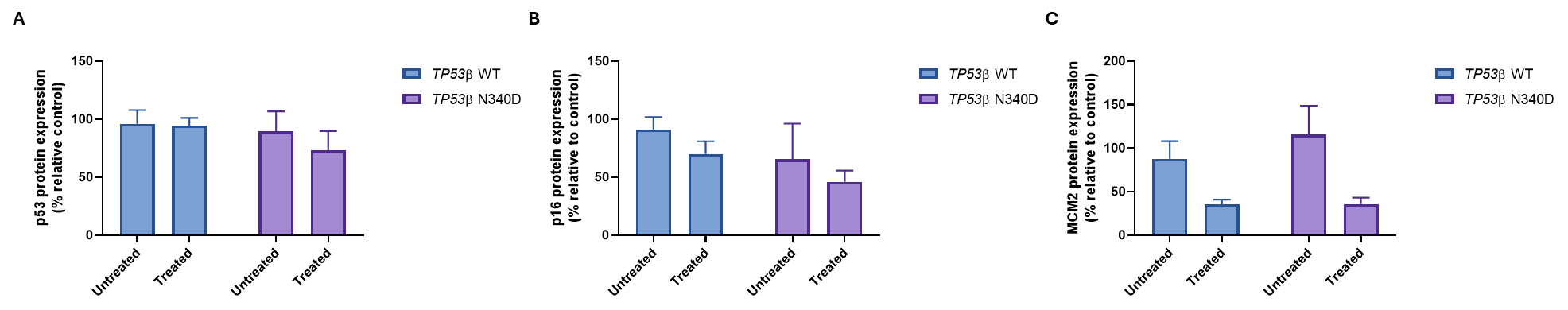


**Figure A.12.** Western blot analysis of p53 **(A)**, p16 **(B)**, and MCM2 **(C)** expression in PBMCs, isolated from *TP53*β WT and *TP53*β N340D control individuals and breast cancer patients, following 1 µM DXR treatment for 72 hours. Data are presented as mean ± SEM (N = 2-8). PBMCs: peripheral blood mononuclear cells; *TP53*: tumour suppressor protein 53; WT: wild type; DXR: doxorubicin.
